# Uptake of Tailored Text Message Smoking Cessation Support in Pregnancy When Advertised on the Internet (MiQuit): Observational Study

**DOI:** 10.2196/jmir.8525

**Published:** 2018-04-19

**Authors:** Joanne L Emery, Tim Coleman, Stephen Sutton, Sue Cooper, Jo Leonardi-Bee, Matthew Jones, Felix Naughton

**Affiliations:** ^1^ Behavioral Science Group Institute of Public Health University of Cambridge Cambridge United Kingdom; ^2^ Division of Primary Care School of Medicine University of Nottingham Nottingham United Kingdom; ^3^ Division of Epidemiology and Public Health School of Medicine University of Nottingham Nottingham United Kingdom; ^4^ School of Health Sciences University of East Anglia Norwich United Kingdom

**Keywords:** smoking cessation, pregnancy, internet, telemedicine, public health, social media

## Abstract

**Background:**

Smoking in pregnancy is a major public health concern. Pregnant smokers are particularly difficult to reach, with low uptake of support options and few effective interventions. Text message–based self-help is a promising, low-cost intervention for this population, but its real-world uptake is largely unknown.

**Objective:**

The objective of this study was to explore the uptake and cost-effectiveness of a tailored, theory-guided, text message intervention for pregnant smokers (“*MiQuit*”) when advertised on the internet.

**Methods:**

Links to a website providing *MiQuit* initiation information (texting a short code) were advertised on a cost-per-click basis on 2 websites (Google Search and Facebook; £1000 budget each) and free of charge within smoking-in-pregnancy webpages on 2 noncommercial websites (National Childbirth Trust and NHS Choices). Daily budgets were capped to allow the Google and Facebook adverts to run for 1 and 3 months, respectively. We recorded the number of times adverts were shown and clicked on, the number of *MiQuit* initiations, the characteristics of those initiating *MiQuit*, and whether support was discontinued prematurely. For the commercial adverts, we calculated the cost per initiation and, using quit rates obtained from an earlier clinical trial, estimated the cost per additional quitter.

**Results:**

With equal capped budgets, there were 812 and 1889 advert clicks to the *MiQuit* website from Google (search-based) and Facebook (banner) adverts, respectively. *MiQuit* was initiated by 5.2% (42/812) of those clicking via Google (95% CI 3.9%-6.9%) and 2.22% (42/1889) of those clicking via Facebook (95% CI 1.65%-2.99%). Adverts on noncommercial webpages generated 53 clicks over 6 months, with 9 initiations (9/53, 17%; 95% CI 9%-30%). For the commercial websites combined, mean cost per initiation was £24.73; estimated cost per additional quitter, including text delivery costs, was £735.86 (95% CI £227.66-£5223.93). Those initiating *MiQuit* via Google were typically very early in pregnancy (median gestation 5 weeks, interquartile range 10 weeks); those initiating via Facebook were distributed more evenly across pregnancy (median gestation 16 weeks, interquartile range 14 weeks).

**Conclusions:**

Commercial online adverts are a feasible, likely cost-effective method for engaging pregnant smokers in digital cessation support and may generate uptake at a faster rate than noncommercial websites. As a strategy for implementing *MiQuit*, online advertising has large reach potential and can offer support to a hard-to-reach population of smokers.

## Introduction

### Background

In developed countries, smoking during pregnancy is a leading preventable cause of adverse prenatal outcomes, including miscarriage [1], stillbirth [2,3], and prematurity [4]. It is also associated with a wide range of infant health problems [5]. In the United Kingdom, around 11% of women are estimated to smoke throughout pregnancy [6], but rates rise considerably with increasing social deprivation [6,7], standing at around 5 times higher in the most deprived women than in the least [7]. Children born to smokers are also more likely to become smokers themselves [8]. Thus, smoking in pregnancy not only puts great financial burden on health services but also perpetuates and exacerbates health inequalities. Reducing its prevalence is a public health priority [9].

Most pregnant smokers want to quit [10], and effective interventions exist to help them [11,12]. Specialist Stop Smoking Services in England offer free pregnancy cessation support with proven efficacy [12]; however, uptake is low [13], with convenience and concerns about being judged reported as barriers to access [14]. In addition to addressing these barriers, research efforts have also been focused on developing effective and cost-effective “distance” alternatives that will appeal to pregnant smokers and be used sufficiently to yield a public health benefit. Self-help cessation support appeals to pregnant smokers [15], and delivering self-help by mobile phone text messaging may be helpful for this group, given its low cost, convenience, anonymity, and wide reach potential, with mobile phone ownership high across the socioeconomic spectrum [16]. Systematic review evidence shows that self-help cessation interventions for pregnant smokers can be effective [17] and that mobile phone–based cessation interventions are effective for nonpregnant smokers [18].

### MiQuit Intervention for Pregnant Smokers

We have developed a low-cost, tailored, text-messaging intervention specifically for pregnant smokers (“*MiQuit*”) [19]. *MiQuit* is feasible to deliver and highly acceptable to pregnant smokers [19], and a recent randomized controlled trial (RCT; n=407) found that offering *MiQuit* in addition to usual care shows promising efficacy and cost-effectiveness [20]. As *MiQuit* is fully automated and user-initiated, women can start using it without the need for any health professional involvement, thus minimizing potential implementation costs. However, little is currently known about the likely real-world uptake of *MiQuit* should this become routinely available to pregnant smokers, or what the best implementation strategies might be to maximize its reach and initiate users into support as cost-effectively as possible. The public health impact of an intervention depends crucially on its real-world uptake, as well as its efficacy, but evaluations of smoking cessation interventions have largely neglected to estimate this [21].

### Using the Internet to Offer Cessation Support

The internet has obvious potential as a tool for reaching pregnant smokers and enrolling them into cessation programs, and evidence suggests that a digital intervention may particularly appeal to smokers offered cessation support through digital media [22]. Interventions can be promoted to potential users on the internet through commercial search-engine and banner-based (pop-up) adverts, as well as noncommercial websites. A previous real-world study in an antenatal setting estimated that 3% to 4% of pregnant smokers initiated *MiQuit* after a brief promotional leaflet was placed into their maternity booking pack without any introduction or endorsement from a health professional [23]. If pregnant women will initiate *MiQuit* after reading a brief leaflet, it seems likely that they may do so after reading an online advert. Offering *MiQuit* to pregnant smokers through search engines, in particular, might reach them earlier in pregnancy than they would typically be targeted in antenatal settings, thus maximizing the benefits of quitting to the fetus. Search-based adverts could also present an opportunity to offer support to women when they are motivated to quit, with cohort evidence suggesting that repeated quit attempts may be made throughout pregnancy [24]. As a tool for recruiting smokers into research trials of digital interventions, studies of nonpregnant groups [25-30] suggest that commercial online advertising can achieve high participant yield rapidly [26,27] and can recruit traditionally hard-to-reach smoker populations at relatively low cost [29,30]. However, although studies typically show similar participant characteristics and retention rates for smokers recruited through online versus traditional means [26-28], with the notable exception of younger age in those recruited via social media [27], others have found lower quitting confidence and lower study completion rates among smokers recruited to trials via the internet [31].

We are aware of only 2 previous studies to explore real-world uptake of digital smoking cessation support, rather than recruitment rates to cessation trials, as a consequence of online advertising, both of which targeted nonpregnant smokers [32,33]. To our knowledge, no published studies have explored uptake of cessation support among pregnant smokers via the internet. In addition, we know little about the characteristics of pregnant smokers who can be encouraged to take up digital interventions over the internet. In this study, therefore, we investigate whether pregnant smokers will initiate the *MiQuit* intervention after seeing paid-for or free online advertising; in addition, we monitor the costs incurred and, using a previously obtained estimate for *MiQuit* efficacy [20], assess the extent to which commercial online advertising might be cost-effective. Finally, we document the extent to which users engage with the support program, including the discontinuation rate, and describe key characteristics, exploring differences between pregnant smokers initiating *MiQuit* via different online routes.

## Methods

### Design

This was an evaluation of the uptake of a digital (text-messaging) intervention advertised on the internet. Uptake rates of *MiQuit* were monitored while it was advertised via 4 concurrent online settings: 2 commercial websites and 2 noncommercial webpage links.

### MiQuit Cessation Support for Pregnant Smokers

*MiQuit* provides a 12-week program of automated, theory-guided, interactive support for quitting smoking in pregnancy, delivered by text message. Support is tailored to 12 baseline user characteristics plus name, gestation, and smoking status, the latter collected at 3 and 7 weeks by text message. Tailoring characteristics include nicotine dependence, partner’s smoking status, and confidence, motivation, and determination to quit [19,20,23]. Women initiate *MiQuit* by texting a short code number. They are then invited to complete 12 baseline tailoring questions, including the option of setting a quit date, either by text or by website. Those tailoring by website must answer all other 11 questions. Those tailoring by text are given the option of answering either 6 or 12 tailoring questions but can stop responding at any point. If no tailoring questions are answered using either route, then generic support is delivered. *MiQuit* delivers 0-2 scheduled daily texts (“push” support), including motivational messages; advice about quit attempt preparation, managing cravings, or trigger situations; and information about fetal development and how smoking affects it. Those setting a quit date receive extra support oriented around their nominated date. Users can access on-demand, “pull” support for combatting cravings (“HELP”), returning to abstinence after a lapse (“SLIP”), or for distraction (“QUIZ”). The support lasts for 12 weeks unless discontinued prematurely by the user sending a “STOP” message. *MiQuit* texts are free to receive. Sending the initiation text and any subsequent texts sent by the user are either free or cost the user’s standard text message rate, depending on their phone “bundle.”

### Web-Based Advertising Campaign

#### Overview of Advertising Methods

With the aim of reaching as many pregnant smokers as possible, we chose 2 commercial advertisers with very large reach potential: Google AdWords [34] (search-based) and Facebook Ads [35] (banner). To identify UK websites most likely to appear as a result of internet searches for smoking-in-pregnancy keywords, 2 search engines (Google and Bing) were used to identify the top webpages returned for the phrase “quit smoking in pregnancy” and close variants. The National Health Service (NHS) website (“NHS Choices”) and the National Childbirth Trust (NCT) website, whose “smoking in pregnancy” webpages [36,37] were consistently close to the top of the search results, agreed to place free-of-charge, text-only links to *MiQuit* on these webpages.

#### Advert Content

Key points made in the adverts were that *MiQuit* is smoking cessation support by text message; *MiQuit* is for pregnant smokers; *MiQuit* is NHS supported; and *MiQuit* is free to receive. Separate adverts were created for each of the 4 online settings, with input from a Patient and Public Involvement representative, keeping the text as similar as possible between adverts given their character or space limits (Multimedia Appendix 1).

#### MiQuit Sign-Up Website

Clicking on any of the 4 adverts led directly to a *MiQuit* sign-up website that provided further information about *MiQuit* and how to initiate it. Each advert led to a separate website clone with a different short code number, enabling us to isolate traffic and initiations from each source. Those wanting to initiate *MiQuit* had to navigate to the “sign-up” page, click on the “sign-up” button, and submit a response to a question asking where they first heard about *MiQuit* (“submissions”). The latter acted as a check that women had not reached the website through other means than our online adverts, such as through the recommendation of a health professional. They were then presented with the short code number on a webpage, with instructions to text the word “QUIT” to the number to begin support. The 4 short codes were not promoted anywhere outside of the 4 cloned websites. To ensure that the websites would not appear in the results of search engines, we added the “disallow” command on the websites’ robots.txt file, which requests Web robots not to scan the websites. This was checked periodically to ensure the most commonly used search engines complied with this request.

#### Commercial Advert Settings

Google AdWords displays a brief, 3-line, text-only advert when advertiser-specified keywords are typed into Google Search. Facebook Ads display a text and image banner advert, unsolicited, to a specified demographic (eg, by age, gender, location, and interests), potentially multiple times per person. We added an image of a pregnant smoker, used elsewhere for promoting *MiQuit* [23], to the text for the Facebook advert. Detailed descriptions of Google and Facebook advertising can be found elsewhere [29,30] but, with both, costs depend on competition from other advertisers. We used a cost-per-click option for both adverts. As we could find no similar studies among pregnant smokers to inform how expenditure would translate into initiations, we set a capped budget of £1000 for each. We restricted both adverts to the United Kingdom, but put no time of day or day of week limits on their scheduling. Google keyword phrases specified were “smoking in pregnancy,” “stop smoking in pregnancy,” “dangers of smoking in pregnancy,” and close variants. Estimated search traffic was relatively low for these; so, broad-match keywords, which permit any combination of the words comprising the phrase, were added to widen reach. On the basis of estimated search traffic and click costs for our keywords (provided by Google), we set a daily budget of £33 for the Google advert, hence a campaign duration of 1 month. For Facebook, we restricted our advert to females aged 16-45 years and specified “pregnancy” and “childbirth” as interests. On the basis of the estimated click costs for our target audience (provided by Facebook), we set a daily budget of £10 for the Facebook advert, hence a campaign duration of 3 months.

#### Free Links

A brief, text-only advert was displayed, permanently for 6 months, on both the NCT and NHS Choices webpage on smoking in pregnancy, under an “external links” section. These had low screen visibility compared with the 2 commercial adverts.

### Procedure

The 4 adverts were run concurrently, beginning late May 2015, either until their budget ended (commercial adverts) or for 6 months (free links). Initiations were permitted up to 3 months after each advert ceased to be shown. The 2 commercial adverts were monitored closely throughout the campaigns. The performance metrics these supply (shown below) were compiled on a weekly basis. Numbers of *MiQuit* submissions, initiations, and discontinuations were compiled weekly for all 4 sources.

### Measures and Analyses

#### Advert Performance and Uptake of *MiQuit*

The 2 commercial advertisers supplied a variety of reach and cost metrics (Textbox 1). The 2 noncommercial websites, NHS Choices and NCT, each provided the number of unique visits to their smoking in pregnancy webpage, where our advert was located. We used Google Analytics on the landing webpages of the *MiQuit* sign-up website for these noncommercial adverts to determine the number of clicks they received.

For all 4 cloned *MiQuit* websites, the *MiQuit* server recorded the number of times the initiation short code was accessed (submissions), whether submissions were from a desktop or mobile phone, and the number of *MiQuit* initiations. As in similar online uptake studies among nonpregnant smokers [32,33], uptake rate was calculated as the percentage of *MiQuit* initiations out of the total number of clicks on an advert to the *MiQuit* sign-up website. Data are presented as frequencies and percentages, with 95% Wilson CIs. For the commercial adverts, we also estimated the number of initiations per individual reached and calculated the cost per initiation. For the purposes of analyses, it was assumed that each advert click, submission, and initiation represented one individual, and that each Google advert impression represented one individual reached. Facebook provided the number of individuals reached by the advert (“people served”), given that an individual can be targeted repeatedly.

#### Estimated Cost-Effectiveness

We estimated the likely incremental cost per additional quitter (also known as the “Incremental Cost-Effectiveness Ratio”) of both initiating women into *MiQuit* via commercial online advertising and delivering the support by summing the mean commercial advert cost and mean cost of sending the texts in this study (£0.035 per text at time of study), divided by the incremental quit rate found in a recent RCT of the *MiQuit* intervention (3.46%) [20]. In this RCT, pregnant smokers (n=407) were recruited from 16 antenatal clinics in England via face-to-face contact and, after responding to tailoring questions by telephone to a researcher, were randomized to receive either *MiQuit* added to usual NHS smoking cessation care or usual care alone. The quit rate for prolonged, biochemically-validated abstinence in the *MiQuit* group was 5.42% (1.96% for usual care) and the odds ratio, adjusted for site and gestation, was 2.70 (95% CI 0.93-9.35) for *MiQuit* over usual care [20]. This is the best estimate yet produced for the likely efficacy of *MiQuit*, although it has limited precision. To determine the impact of uncertainty, we bootstrapped 1000 times our incremental quit rate and cost to estimate the 95% CIs for the cost per additional quitter [38]. In addition, there was a fixed annual running cost, shared across all users, of approximately £760, consisting of a virtual reply number (£99), Web hosting with domain name (£240), and short code (£420). This was not included in the cost-effectiveness analysis as a per-person cost could not be calculated: the annual number of users is currently an unknown quantity.

#### User Engagement and Characteristics

*MiQuit* server data were used to assess engagement with the support program, including rates of tailoring question completion, quit date setting, use of “pull-support” features, and discontinuations (sending a “STOP” message). Data are presented as frequencies and percentages, with 95% Wilson CIs presented for key measures (discontinuation rate and quit date setting). Key behavioral characteristics of those initiating *MiQuit* were taken from their responses to the tailoring questions, answered by Web or text. Characteristics were compared between those initiating *MiQuit* via different online sources, where numbers permitted, using Mann-Whitney *U* tests (continuous data) and Fisher exact tests (categorical data).

**Advert reach and cost metrics supplied by the 2 commercial advertisers.**

- Impressions—number of times the advert was displayed.
- People served (Facebook only)—number of individuals the advert was displayed to.
- Impression share (Google only)—proportion of times the advert was displayed when a relevant keyword search was made. This shows the number of impressions achievable given an unlimited budget.
- Clicks—number of times the advert was clicked on. A click took the user directly to the *MiQuit* sign-up website, so the number of clicks equates to the number of website visits.
- Cost per click—mean cost incurred for a single advert click.
- Proportion of impressions and clicks by device type (desktop or mobile).
- Mean screen position (Google only)—mean location of advert impressions on the Google search results page (1=top of screen).

### Ethical Approval

Advice was sought from the National Research Ethics Central Queries Service as to whether the study should be classed as research requiring ethical review, and they confirmed that no ethical review was required. The study was conducted in accordance with the ethical principles that have their origin in the Declaration of Helsinki, 1996; the Principles of Good Clinical Practice; and the Department of Health Research Governance Framework for Health and Social Care, 2005. Participants were able to withdraw from the *MiQuit* support at any time.

## Results

### Advert Performance and Uptake of MiQuit

Most commercial advert clicks came from mobile devices rather than desktops (Google: 560/812, 69.0%; Facebook: 1883/1889, 99.68%). Of those who accessed the initiation short code, 94.1% (301/320) did so from a mobile device (Google: 110/121, 90.9%; Facebook: 184/187, 98.4%; NHS Choices: 1/4, 25%; NCT: 6/8, 75%).

**Figure 1:**
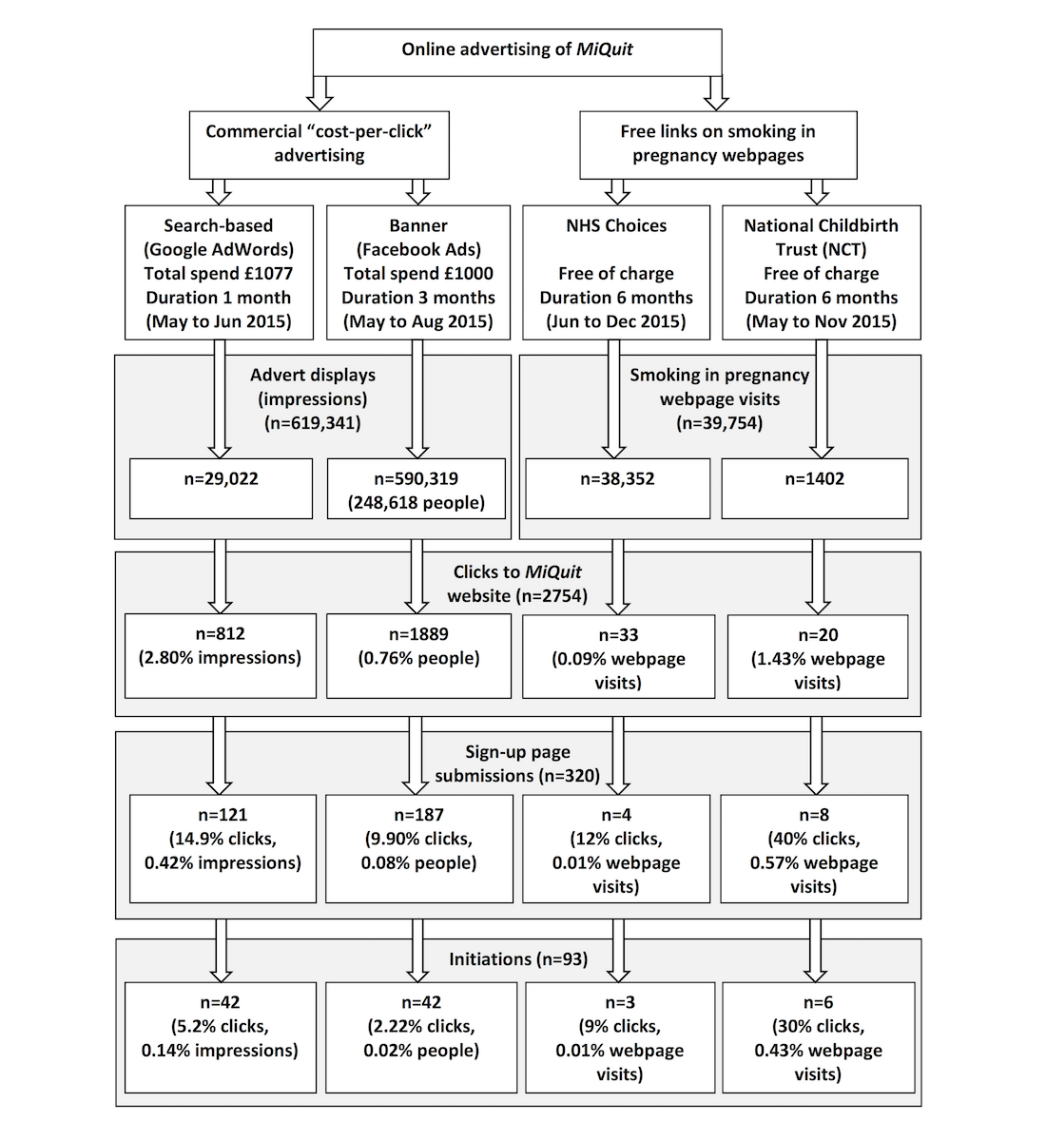
Flow diagram: *MiQuit* advert reach and initiation of support.

Figure 1 shows the flow of the targeted populations, through each online route, into initiation of *MiQuit* support. The Google advert was shown 29,022 times in its 1-month duration. Given our impression share of 70% for mobile-based searches and 50% for desktop-based searches, we estimated that over 46,000 Google searches had been made for our keywords, in the United Kingdom, during this time. The mean position of the Google advert from the top of the screen was 1.0 for mobiles and 1.2 for desktops (highly visible). The Facebook advert was shown to 248,618 broadly targeted women during its 3-month duration (mean 2.4 times each) and also had high visibility on screen (Multimedia Appendix 1). In 6 months, approximately 40,000 unique visits were made to the NHS Choices and NCT smoking-in-pregnancy webpages containing the *MiQuit* link, but the proportions who scrolled down to where the links were placed on these is unknown.

In total, 2754 individuals clicked on 1 of the 4 adverts to the *MiQuit* sign-up website (Google n=812, Facebook n=1889, NHS Choices n=33, NCT n=20). For Google, assuming 1 advert impression per person, this amounted to 2.80% of those served the advert (812/29,022) and, for Facebook, 0.76% of those served the advert (1889/248,618). For the NHS Choices and NCT websites, the numbers of clicks on the *MiQuit* links amounted to 0.09% (33/38,352) and 1.43% (20/1402), respectively, of the numbers of unique visits to the webpages containing the link.

*MiQuit* was initiated by 93 individuals in total, with the 2 commercial campaigns each yielding 42 initiations and the free links 9 in total. For our uptake rate calculation, the percentage who subsequently initiated *MiQuit* after clicking on an advert to the *MiQuit* website was 3.38% (93/2754, 95% CI 2.76%-4.12%); Google 5.2% (42/812, 95% CI 3.9%-6.9%), Facebook 2.22% (42/1889, 95% CI 1.65%-2.99%); NHS Choices 9% (3/33; 95% CI 3%-24%), NCT 30% (6/20; 95% CI 15%-52%). One in 691 Google advert impressions resulted in a *MiQuit* initiation (1 in 5919 women targeted by Facebook). One in 12,784 visits to the NHS Choices webpage, and 1 in 234 visits to the NCT webpage, resulted in an initiation.

### Commercial Advertising Costs and Estimated Cost-Effectiveness

The Facebook campaign cost £1000, whereas the Google campaign cost £1077, including £75 credited free to our account. Table 1 shows a breakdown of these costs in terms of how far *MiQuit* was accessed or activated. The mean cost per advert click to the *MiQuit* website was £1.33 for the Google advert and £0.53 for the Facebook advert. Both campaigns yielded equal initiations for their budget. Cost per *MiQuit* initiation was £25.64 via Google, £23.81 via Facebook, and £24.73 across both campaigns. Table 1 also shows the estimated cost per additional quitter of initiating pregnant smokers into *MiQuit* via commercial online advertising. Using the cost per initiation of the commercial adverts (£24.73) plus the mean cost of sending the *MiQuit* texts to those initiating support here (£2.73) gave a point estimate of £793.64 per additional quitter to both initiate and deliver the support. The mean cost per additional quitter from the bootstrap was £735.86 (95% CI £227.66-£5223.93).

### Engagement and Disengagement With MiQuit

A total of 53 of the 93 initiators in this study (57%, 95% CI 47%-67%) set at least 1 quit date with *MiQuit* during the program, including at baseline. Moreover, 63 (68%) of the initiators chose to answer all 12 tailoring questions by website (with quit date noncompulsory). Of those who chose to answer by text message instead (n=27), 16 (59%) responded to at least the first 6 questions. Only 3 initiators (3%) answered no tailoring questions and received generic support. “Pull” support features (“HELP,” “SLIP,” and “QUIZ” requests) were used by 35 (38%) initiators.

A total of 34 of the 93 initiators in this study (37%, 95% CI 27%-47%) stopped the 86-day program prematurely (mean days into program 18.6, SD 21.3). Although not formally tested, discontinuation rates, tailoring question completion rates, and use of interactive program features did not appear to differ between those taking up *MiQuit* via the 4 different online routes.

### Characteristics of Those Initiating MiQuit

Table 2 shows key characteristics of those who initiated *MiQuit* via online advertising (n=93) and statistically compares those who initiated *MiQuit* via Google versus Facebook; numbers were insufficient to compare data from the 2 free links (shown combined). Readiness to quit smoking appeared high among initiators in this study, with 70 out of 93 (78%) seriously planning to quit within the next 2 weeks. Gestation differed substantially between women who initiated support via Google versus Facebook; no other characteristic differed significantly between them. Those from Google were typically very early in pregnancy, with 49% reporting a baseline gestation of 4 or 5 weeks (median 5 weeks; interquartile range [IQR] 10); those from Facebook were distributed more widely across pregnancy (median 16 weeks, IQR 14, Mann-Whitney *U* test *P*<.001).

**Table 1:** Costs and estimated cost-effectiveness of the commercial online adverts.

Advertising campaign	Cost per:			Estimated cost per additional quitter^a^
	Advert click, leading to the *MiQuit* website	Short code obtained	*MiQuit* initiation	
Google AdWords (spend £1077)^b^	£1.33 (n=812)	£8.90 (n=121)	£25.64 (n=42)	£741.04
Facebook Ads (spend £1000)	£0.53 (n=1889)	£5.35 (n=187)	£23.81 (n=42)	£688.15
Both campaigns (spend £2077)^b^	£0.77 (n=2701)	£6.74 (n=308)	£24.73 (n=84)	£714.74

^a^On the basis of an incremental quit rate of 3.46% in the *MiQuit* randomized controlled trial (RCT) [20] (prolonged, validated abstinence).

^b^Including £75 credited free to our account by Google as a welcome offer.

**Table 2:** Baseline characteristics of pregnant smokers initiating *MiQuit* via online advertising.

Baseline characteristic		Total (n=93)	Google AdWords (n=42)	Facebook Ads (n=42)	Free links (total)^a^ (n=9)	*P* value^b^
**Gestation (weeks)**						**<.001**
	Mean (SD)	12.3 (8.5)	9.1 (7.2)	16.7 (8.5)	7.1 (4.1)	
	Median (1st Q, 3rd Q)	9.5 (5, 18)	5 (4, 14)	16 (10, 24)	6 (4, 8)	
	Min, max	2, 32	2, 28	4, 32	4, 17	
	Valid n (%)	90 (96.8)	41 (97.6)	40 (95.2)	9 (100)	
**Are you seriously planning to quit? n (%)**						**.30**
	Within the next 2 weeks	70 (78)	35 (83)	28 (72)	7 (78)	
	Within the next 30 days	15 (17)	6 (14)	7 (18)	2 (22)	
	Within the next 3 months	3 (3)	0 (0)	3 (8)	0 (0)	
	No	2 (2)	1 (2)	1 (2)	0 (0)	
	Valid n (%)	90 (97)	42 (100)	39 (93)	9 (100)	
**Confidence to quit for remainder of pregnancy, n (%)**						**.36**
	Not at all	21 (26)	6 (17)	13 (35)	2 (25)	
	A little	18 (23)	8 (23)	7 (19)	3 (38)	
	Moderately	29 (36)	15 (43)	12 (32)	2 (25)	
	Very much	11 (14)	6 (17)	4 (11)	1 (13)	
	Extremely	1 (1)	0 (0)	1 (3)	0 (0)	
	Valid n (%)	80 (86)	35 (83)	37 (88)	8 (89)	
**Cigarettes per day now, n (%)**						**.33**
	1-3	6 (8)	5 (14)	1 (3)	0 (0)	
	4-5	16 (20)	6 (17)	9 (25)	1 (13)	
	6-10	23 (29)	9 (26)	12 (33)	2 (25)	
	11-15	19 (24)	9 (26)	8 (22)	2 (25)	
	16-20	12 (15)	4 (11)	6 (17)	2 (25)	
	21+	3 (4)	2 (6)	0 (0)	1 (13)	
	Valid n (%)	79 (85)	35 (83)	36 (86)	8 (89)	
**Heaviness of Smoking Index^c^** **, n (%)**						**.24**
	Very low	19 (24)	6 (17)	12 (33)	1 (13)	
	Low to moderate	33 (42)	18 (51)	12 (33)	3 (38)	
	Moderate	24 (30)	9 (26)	12 (33)	3 (38)	
	High	3 (4)	2 (6)	0 (0)	1 (13)	
	Valid n (%)	79 (85)	35 (83)	36 (86)	8 (89)	
**Partner’s smoking status, n (%)**						**.30**
	Smoker	43 (61)	16 (53)	22 (69)	5 (63)	
	Nonsmoker or no partner	27 (39)	14 (47)	10 (31)	3 (38)	
	Valid n (%)	70 (75)	30 (71)	32 (76)	8 (89)	

^a^Data for NCT (n=6) and NHS Choices (n=3) were combined because of small numbers.

^b^Facebook vs Google *P* value. Tested via Mann-Whitney *U* (continuous) or Fisher Exact test (frequencies).

^c^Heaviness of Smoking Index was based on the sum of scores from 2 items of the Fagerström Test of Cigarette Dependence [39]: cigarettes per day (1-10=score of 0, 11-20=1, 21-30=2, >30=3) and time to first cigarette after waking (>1 hour=0, 31-60 min=1, 6-30 min=2, within 5 min=3). A combined score of 0-2=very low dependence, 3=low to moderate dependence, 4=moderate dependence, and 5-6=high dependence.

## Discussion

### Principal Findings

When a low-cost, text messaging, pregnancy smoking cessation support program (*MiQuit*) was advertised via the internet, with no other form of promotion or recommendation, an overall uptake (initiation) rate of 3.4% was seen among those who clicked on any of the 4 adverts to the *MiQuit* website. Commercial adverts, which yielded the vast majority of initiations in this study, cost, on average, £24.73 per initiation. Although the initiation rate was higher among those who reached the *MiQuit* website via free webpage links, the total number of initiations generated through these was much lower than the number of initiations generated by commercial advertising and occurred over a longer time frame. Behavioral characteristics appeared similar for those initiating *MiQuit* from different online sources. User engagement appeared high as over half of initiators set a quit date with the system, and approximately two-thirds continued with *MiQuit* until the end of the 12-week program.

### Strengths and Limitations

To our knowledge, this is the first study to explore the feasibility of using free and paid-for online advertising to reach pregnant smokers and promote their uptake of cessation support. We have shown that a significant minority of pregnant smokers are willing to initiate an automated text messaging intervention when offered this via the internet; given the high reach of the internet, this could translate into substantial numbers of pregnant smokers supported to quit. Unlike many previous evaluations of online advertising [25], we were able to trace the source of all those who arrived at the *MiQuit* website and all who initiated support, allowing us to compare uptake rates for different online strategies. By quantifying each step of the uptake process, from viewing an advert through to initiating support, we have explored a spectrum of engagement [40] with the offer of support and identified steps where barriers to uptake might be removed. Importantly, we tracked individuals’ behavior beyond support initiation, investigating their engagement with the program (quit date setting, use of “pull” support messages, tailoring question completion) as well as discontinuations (sending a “STOP” message). We were also able to use previous RCT data, with assumptions, to estimate the likely cost-effectiveness of *MiQuit* if implemented via commercial online adverts.

There are several limitations to this study, including some general challenges for real-world uptake studies. As with many studies recruiting people to interventions without person involvement, we cannot be sure that those who took up support were the intended group (ie, pregnant smokers), although our adverts and website made it clear that *MiQuit* is for pregnant smokers and almost all provided their gestation. It is also possible that adverts were clicked on out of curiosity by those who were neither pregnant nor smoking, particularly among women targeted by banner adverts on Facebook, or that adverts were passed on to others (eg, friends) by those originally targeted. In addition, it proved difficult to estimate the numbers of pregnant smokers exposed to each advert as a denominator: the number of pregnant smokers shown our Facebook advert is unknown and it is probable that not all Google searches for our keywords were made by pregnant smokers. Nonetheless, the high number of keyword searches made within the United Kingdom in a month (over 46,000) suggests that there may be many opportunities for engaging with this population on the internet. Given the low visibility of the 2 free links, our use of the number of visits to the webpages on which these were placed as a denominator may have greatly underestimated the proportions initiating *MiQuit* out of those exposed. Difficulties in specifying accurate and comparable denominators have been noted previously in uptake studies [23,41]. However, to health providers, having accurate information on the numbers of *MiQuit* initiations and on the cost of providing this is of the greatest importance.

It is important to be aware of assumptions inherent to the cost-effectiveness estimates presented. Our cost-effectiveness analyses assumed that, when initiated via the internet, *MiQuit* would have a similar quit rate to that observed in women recruited to an RCT from antenatal clinics. However, we found differences in characteristics between women in this study and those recruited to the RCT (discussed below), and it is possible that the intervention could vary in effectiveness for different groups of women. Both Facebook and Google had relatively opaque criteria used to determine their advertising charges; there is no guarantee that similar charges would be made for identical adverts in the future. Charges depend on concurrent competition from other advertisers, so are unlikely to replicate exactly from one campaign to another even if all other parameters are held constant. It is probably best to view our cost-effectiveness estimates as indicative rather than definitive; however, optimization of this type of “programmatic” advertising would likely reduce costs significantly.

### Findings in Context

Compared with pregnant smokers who were recruited to a large RCT of *MiQuit* via antenatal clinics in a previous study [20], women who initiated *MiQuit* via online advertising in this study had higher readiness to quit, with 78% (vs 32% RCT) seriously planning to quit within the next 2 weeks and 57% (vs 19% RCT) sending a quit date to *MiQuit* during the program, including at baseline. This suggests that online initiators may be more likely to make a quit attempt [42,43]. Conversely, online initiators appeared to be more nicotine-dependent than RCT recruits, with 34% (vs 14% RCT) classed as “moderate” or “high” dependence, and this appears to be a key determinant of failure to quit during pregnancy [43-46]. It is, therefore, possible that quit rates might be lower among individuals who engage with *MiQuit* via the internet compared with those who did so after being recruited to a trial in an NHS setting, but this is currently speculative. Others have not found heavier smoking among online recruits [31].This is an important avenue for future evaluation: *MiQuit* could have different effects depending on how it is implemented and, therefore, who makes use of it. Previous research has found high readiness to quit in smokers recruited to RCTs by search-based [29], but not banner, online advertising methods [28]; however, in this study, readiness to quit was similar between those initiating *MiQuit* via Google and Facebook.

A total of 37% of initiators in this study stopped the 12-week program prematurely (sent a “STOP” message). This was notably higher than the discontinuation rate among trial participants, where 13% randomized to the *MiQuit* condition discontinued the support [20], though more similar to the discontinuation rate when *MiQuit* was offered in an NHS real-world context, by leaflet (46%) [23]. It is possible that those receiving *MiQuit* as part of an RCT felt obliged to continue with it because of being a trial participant and receiving human contact as part of their involvement. Previous *MiQuit* research highlights that discontinuations are made for a variety of reasons, most of which are not related to irritation or dissatisfaction [19], and a separate study indicates that discontinuation can be an indicator of increased engagement in smoking cessation behavior [47]. In other ways, online initiators appeared more engaged with *MiQuit* than RCT participants, with 3 times as many sending a quit date to the system and almost twice as many (38% vs 21%) sending a “pull” support message.

Although our Facebook advert generated activations throughout pregnancy, those who initiated *MiQuit* via Google were often early in gestation (around 50% within their first 5 weeks). Adverts attached to internet search engines may, therefore, be a useful way to reach women when they are first pregnant and looking for support, and could potentially maximize health benefits by encouraging abstinence for more of their pregnancy. Currently, the earliest used cessation interventions tend to target pregnant smokers at their antenatal booking appointment, at around 8-12 weeks’ gestation.

We have shown that uptake of *MiQuit* via online advertising is feasible; our previous real-world study showed uptake of *MiQuit* to be feasible when offered via leaflets in maternity booking packs without health professional promotion. This suggests that there are promising routes to initiating pregnant smokers into support systems such as *MiQuit*, without the need for health professional involvement, in both clinical and nonclinical settings. We are aware of no other studies that have investigated using the internet for offering real-world cessation support to pregnant smokers, although 2 previous studies have explored this among nonpregnant smokers in the United States [32,33]. Our overall uptake rate (3.4%) was lower than that found among all smokers offered a national Web-based cessation program via commercial search-based and banner online adverts (6.8%) [32]; however, it was similar to the uptake rate found when banner adverts were used to promote Web-based cessation support specifically to Latino smokers, another hard-to-reach group (2.8%) [33]. Our average cost per initiation (£24.73) compares favorably with costs reported to initiate general smokers into real-world digital cessation support commercially via the internet (mean $35) [32] and very favorably with costs reported to initiate a hard-to-reach group via online banner methods (mean $209.34) [33]. Facebook may be more cost-effective than other banner-based methods for targeting specific populations, given that adverts can be restricted to a particular demographic [48].

Using an efficacy estimate of *MiQuit* from a previous RCT, we estimated a cost per additional quitter of £735.86 to initiate pregnant smokers into *MiQuit* through paid adverts, including text message delivery costs but excluding development costs. We did not include a formal cost-utility analysis here, and the caveats discussed in our limitations must be noted, but this is encouraging compared with costs reported for other smoking cessation interventions that are effective and cost-effective in pregnancy. For example, financial incentives are highly cost-effective, with a cost per additional quitter of £1127 [49].

Of those who clicked to the *MiQuit* website, a much greater percentage (11.6%) obtained the short code to initiate *MiQuit* than subsequently texted it to do so (3.4%). Obtaining the short code required a number of extra steps after clicking on an advert and landing on the *MiQuit* website, suggesting that these women were serious about taking up *MiQuit*. There may thus be potential for increasing uptake substantially, at no extra advertising cost, by ameliorating the drop-off between clicking to accept the support and texting to initiate it. Having to text a short code may be a barrier to initiating support for a number of reasons, including lack of credit among pay-as-you-go phone users, suspicion of hidden charges, and needing to act outside of the website; enabling women to sign up anonymously without the need to text a short code might increase uptake. Website content, tone, and appearance are also potential targets; clearly labeling the website as an NHS service might also reduce women’s barriers to sending an initiation text.

A definitive evaluation is planned for *MiQuit*. If it is shown to be effective, as our earlier trial suggests is likely [20], then an assessment of its efficacy in an online setting may be warranted. In this study, free-of-charge webpage links yielded relatively few initiations but might have performed better if given greater visibility. High initiation rates were found for women clicking from these links, suggesting that they were well targeted despite having lower reach than the commercial adverts, and pregnant smokers may have been more likely to initiate *MiQuit* if reaching it from a recognized health source. There may therefore be scope for future work to promote *MiQuit* via such websites. Future work could aim to minimize search engine–based advertising costs by investigating which specific “smoking in pregnancy” keyword phrases are associated with support initiations; this is possible if support is initiated by a webpage click rather than an external action such as texting a short code. Finally, it is important to establish whether uptake of text-based cessation support among pregnant smokers affects their uptake of traditional cessation support or whether it attracts those who would otherwise try to quit alone, if at all.

### Conclusions

Commercial online advertising appears to be a promising method for initiating pregnant smokers into text message–based cessation support. Free and commercial adverts prompting pregnant smokers to click to a sign-up website resulted in an initiation rate of 3.4%. Search-based commercial advertising was able to reach women earlier in pregnancy than interventions delivered in clinical settings seem able to achieve, and those who initiated support in this study had high readiness to quit. Commercial online advertising to pregnant smokers is likely to be cost-effective and can probably be made more so. Given that pregnant smokers’ uptake of traditional support is low, it is important to find successful strategies for offering them effective alternatives.

## Appendix

Multimedia Appendix 1 Advert appearance on screen.

## Abbreviations

NHS: National Health Service
NCT: National Childbirth Trust
RCT: randomized controlled trial

## References

[1] Pineles BL, Park E, Samet JM (2014). Systematic review and meta-analysis of miscarriage and maternal exposure to tobacco smoke during pregnancy. Am J Epidemiol.

[2] Flenady V, Koopmans L, Middleton P, Frøen JF, Smith GC, Gibbons K, Coory M, Gordon A, Ellwood D, McIntyre HD, Fretts R, Ezzati M (2011). Major risk factors for stillbirth in high-income countries: a systematic review and meta-analysis. Lancet.

[3] Högberg L, Cnattingius S (2007). The influence of maternal smoking habits on the risk of subsequent stillbirth: is there a causal relation?. BJOG.

[4] Moore E, Blatt K, Chen A, Van Hook J, DeFranco EA (2016). Relationship of trimester-specific smoking patterns and risk of preterm birth. Am J Obstet Gynecol.

[5] Cnattingius S (2004). The epidemiology of smoking during pregnancy: smoking prevalence, maternal characteristics, and pregnancy outcomes. Nicotine Tob Res.

[6] NHS Digital (2016). http://content.digital.nhs.uk/catalogue/PUB20219.

[7] NHS Digital (2012). http://content.digital.nhs.uk/catalogue/PUB08694.

[8] Leonardi-Bee J, Jere ML, Britton J (2011). Exposure to parental and sibling smoking and the risk of smoking uptake in childhood and adolescence: a systematic review and meta-analysis. Thorax.

[9] HM Government (2011). https://www.gov.uk/government/uploads/system/uploads/attachment_data/file/213757/dh_124960.pdf.

[10] Ussher M, West R, Hibbs N (2004). A survey of pregnant smokers' interest in different types of smoking cessation support. Patient Educ Couns.

[11] Chamberlain C, O'Mara-Eves A, Oliver S, Caird JR, Perlen SM, Eades SJ, Thomas J (2013). Psychosocial interventions for supporting women to stop smoking in pregnancy. Cochrane Database Syst Rev.

[12] Bauld L, Bell K, McCullough L, Richardson L, Greaves L (2010). The effectiveness of NHS smoking cessation services: a systematic review. J Public Health (Oxf).

[13] NHS Digital (2016). http://content.digital.nhs.uk/catalogue/PUB21162.

[14] Ussher M, Etter JF, West R (2006). Perceived barriers to and benefits of attending a stop smoking course during pregnancy. Patient Educ Couns.

[15] Ussher M, West R, Hibbs N (2004). A survey of pregnant smokers' interest in different types of smoking cessation support. Patient Educ Couns.

[16] Office for National Statistics (2017). https://www.ons.gov.uk/peoplepopulationandcommunity/personalandhouseholdfinances/expenditure/datasets/percentageofhouseholdswithdurablegoodsbyincomegroupandhouseholdcompositionuktablea46.

[17] Naughton F, Prevost AT, Sutton S (2008). Self-help smoking cessation interventions in pregnancy: a systematic review and meta-analysis. Addiction.

[18] Whittaker R, McRobbie H, Bullen C, Rodgers A, Gu Y (2016). Mobile phone-based interventions for smoking cessation. Cochrane Database Syst Rev.

[19] Naughton F, Prevost AT, Gilbert H, Sutton S (2012). Randomized controlled trial evaluation of a tailored leaflet and SMS text message self-help intervention for pregnant smokers (MiQuit). Nicotine Tob Res.

[20] Naughton F, Cooper S, Foster K, Emery J, Leonardi-Bee J, Sutton S, Jones M, Ussher M, Whitemore R, Leighton M, Montgomery A, Parrott S, Coleman T (2017). Large multi-centre pilot randomized controlled trial testing a low-cost, tailored, self-help smoking cessation text message intervention for pregnant smokers (MiQuit). Addiction.

[21] Marcano Belisario JS, Bruggeling MN, Gunn LH, Brusamento S, Car J (2012). Interventions for recruiting smokers into cessation programmes. Cochrane Database Syst Rev.

[22] Balmford J, Borland R, Benda P, Howard S (2013). Factors associated with use of automated smoking cessation interventions: findings from the eQuit study. Health Educ Res.

[23] Naughton F, Cooper S, Bowker K, Campbell K, Sutton S, Leonardi-Bee J, Sloan M, Coleman T (2015). Adaptation and uptake evaluation of an SMS text message smoking cessation programme (MiQuit) for use in antenatal care. Br Med J Open.

[24] Pickett KE, Wakschlag LS, Dai L, Leventhal BL (2003). Fluctuations of maternal smoking during pregnancy. Obstet Gynecol.

[25] Lane TS, Armin J, Gordon JS (2015). Online recruitment methods for web-based and mobile health studies: a review of the literature. J Med Internet Res.

[26] Heffner JL, Wyszynski CM, Comstock B, Mercer LD, Bricker J (2013). Overcoming recruitment challenges of web-based interventions for tobacco use: the case of web-based acceptance and commitment therapy for smoking cessation. Addict Behav.

[27] Frandsen M, Walters J, Ferguson SG (2014). Exploring the viability of using online social media advertising as a recruitment method for smoking cessation clinical trials. Nicotine Tob Res.

[28] Stanczyk NE, Bolman C, Smit ES, Candel MJ, Muris JW, de Vries H (2014). How to encourage smokers to participate in web-based computer-tailored smoking cessation programs: a comparison of different recruitment strategies. Health Educ Res.

[29] Gordon JS, Akers L, Severson HH, Danaher BG, Boles SM (2006). Successful participant recruitment strategies for an online smokeless tobacco cessation program. Nicotine Tob Res.

[30] Ramo DE, Rodriguez TM, Chavez K, Sommer MJ, Prochaska JJ (2014). Facebook recruitment of young adult smokers for a cessation trial: methods, metrics, and lessons learned. Internet Interv.

[31] Frandsen M, Thow M, Ferguson SG (2016). The effectiveness of social media (Facebook) compared with more traditional advertising methods for recruiting eligible participants to health research studies: a randomized, controlled clinical trial. JMIR Res Protoc.

[32] Graham AL, Milner P, Saul JE, Pfaff L (2008). Online advertising as a public health and recruitment tool: comparison of different media campaigns to increase demand for smoking cessation interventions. J Med Internet Res.

[33] Graham AL, Fang Y, Moreno JL, Streiff SL, Villegas J, Muñoz RF, Tercyak KP, Mandelblatt JS, Vallone DM (2012). Online advertising to reach and recruit Latino smokers to an internet cessation program: impact and costs. J Med Internet Res.

[34] Google Google AdWords.

[35] Facebook.

[36] NHS Choices http://www.nhs.uk/conditions/pregnancy-and-baby/pages/smoking-pregnant.aspx.

[37] National Childbirth Trust https://www.nct.org.uk/pregnancy/smoking-during-pregnancy.

[38] Glick HA, Doshi JA, Sonnad SS, Polsky D (2014). Economic Evaluation in Clinical Trials. 2nd ed.

[39] Heatherton TF, Kozlowski LT, Frecker RC, Fagerström KO (1991). The Fagerström Test for nicotine dependence: a revision of the Fagerström Tolerance Questionnaire. Br J Addict.

[40] Platt T, Platt J, Thiel DB, Kardia SL (2016). Facebook advertising across an engagement spectrum: a case example for public health communication. JMIR Public Health Surveill.

[41] Graham AL, Bock BC, Cobb NK, Niaura R, Abrams DB (2006). Characteristics of smokers reached and recruited to an internet smoking cessation trial: a case of denominators. Nicotine Tob Res.

[42] Vangeli E, Stapleton J, Smit ES, Borland R, West R (2011). Predictors of attempts to stop smoking and their success in adult general population samples: a systematic review. Addiction.

[43] Emery JL, Sutton S, Naughton F (2017). Cognitive and behavioral predictors of quit attempts and biochemically-validated abstinence during pregnancy. Nicotine Tob Res.

[44] Schneider S, Huy C, Schütz J, Diehl K (2010). Smoking cessation during pregnancy: a systematic literature review. Drug Alcohol Rev.

[45] Vaz LR, Leonardi-Bee J, Aveyard P, Cooper S, Grainge M, Coleman T, SNAP trial team (2014). Factors associated with smoking cessation in early and late pregnancy in the smoking, nicotine, and pregnancy trial: a trial of nicotine replacement therapy. Nicotine Tob Res.

[46] Riaz M, Lewis S, Coleman T, Aveyard P, West R, Naughton F, Ussher M (2016). Which measures of cigarette dependence are predictors of smoking cessation during pregnancy? Analysis of data from a randomized controlled trial. Addiction.

[47] Balmford J, Borland R (2014). How do smokers use a smoking cessation text messaging intervention?. Nicotine Tob Res.

[48] Capurro D, Cole K, Echavarría MI, Joe J, Neogi T, Turner AM (2014). The use of social networking sites for public health practice and research: a systematic review. J Med Internet Res.

[49] Boyd KA, Briggs AH, Bauld L, Sinclair L, Tappin D (2016). Are financial incentives cost-effective to support smoking cessation during pregnancy?. Addiction.

