# Supplementary material for: Uptake of Tailored Text Message Smoking Cessation Support in Pregnancy When Advertised on the Internet (MiQuit): Observational Study
Source: J Med Internet Res. 2018 Apr 19;20(4):e146. doi: 10.2196/jmir.8525 (PMC5934538; doi:10.2196/jmir.8525)
Supplement: Multimedia Appendix 1 [file jmir_v20i4e146_app1.pdf]

Google AdWords

We created the following text-only advert to fit within the strict character limits of Google AdWords (limits apply to each line of text). The advert appeared as below, with slight variations depending on screen position (black border added). Mean screen position was 1.2 for desktops and 1.0 for mobile devices.

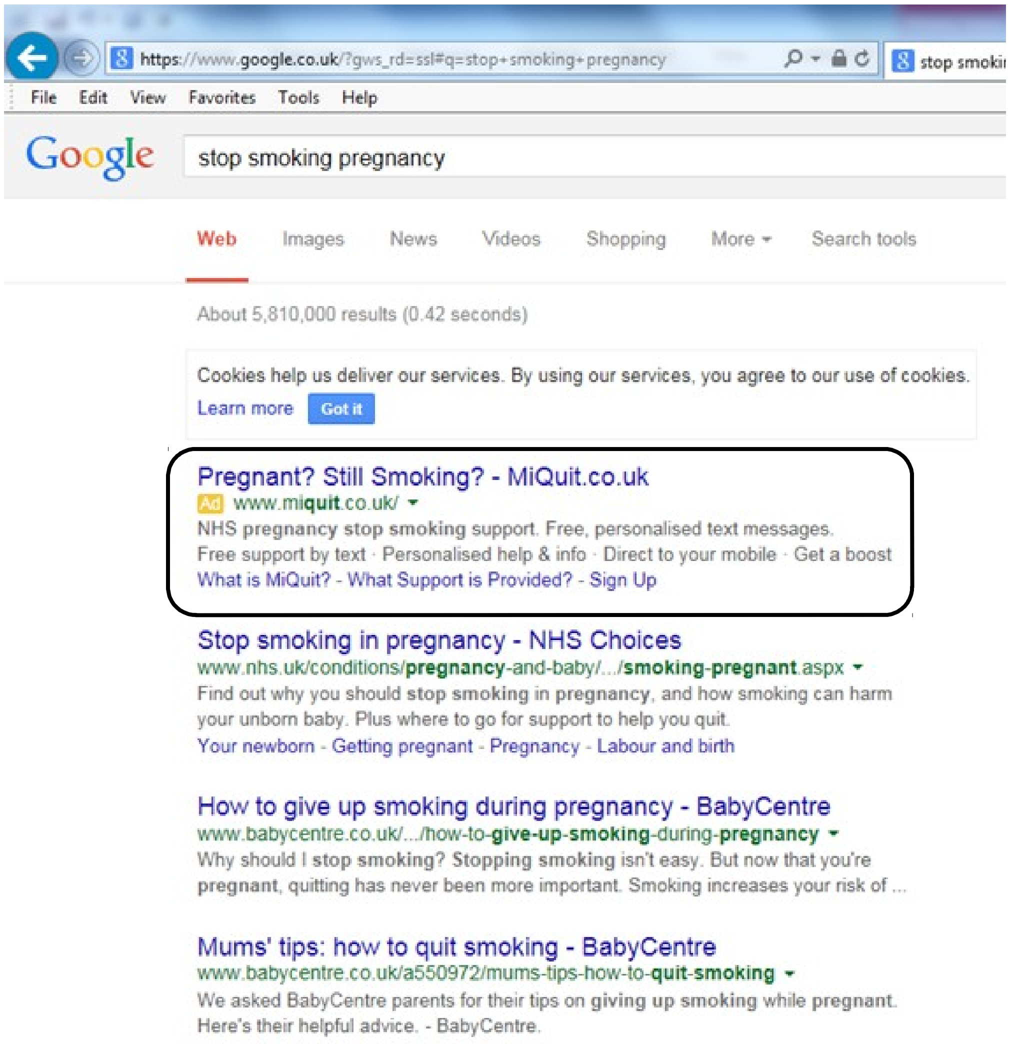

Facebook Ads

For the Facebook advert, we added an image of a pregnant smoker used previously to promote MiQuit.[23] The header used for our Google advert ('Pregnant? Still Smoking?') was rejected by Facebook for referring to personal characteristics of those targeted, and was therefore amended to 'Help for Pregnant Smokers'. Advert appearance varied depending on the device used, with additional text (shown below the image) appearing on mobile devices. The advert appeared as below on mobile devices (black border added).

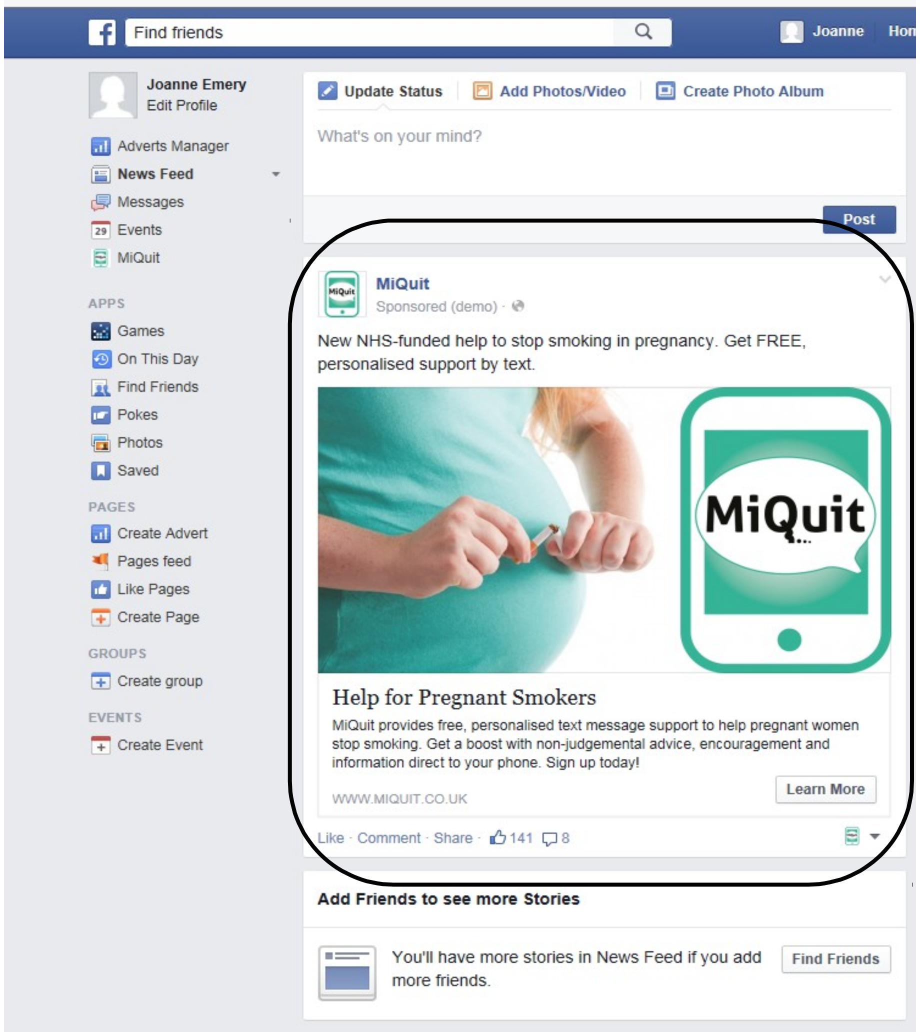

NHS Choices ("Stop Smoking in Pregnancy" Web page)

NHS Choices placed the following text-only link to the MiQuit website under an "External links" section situated towards the bottom of their "Stop Smoking in Pregnancy" Web page. The link appeared as below (black border added).

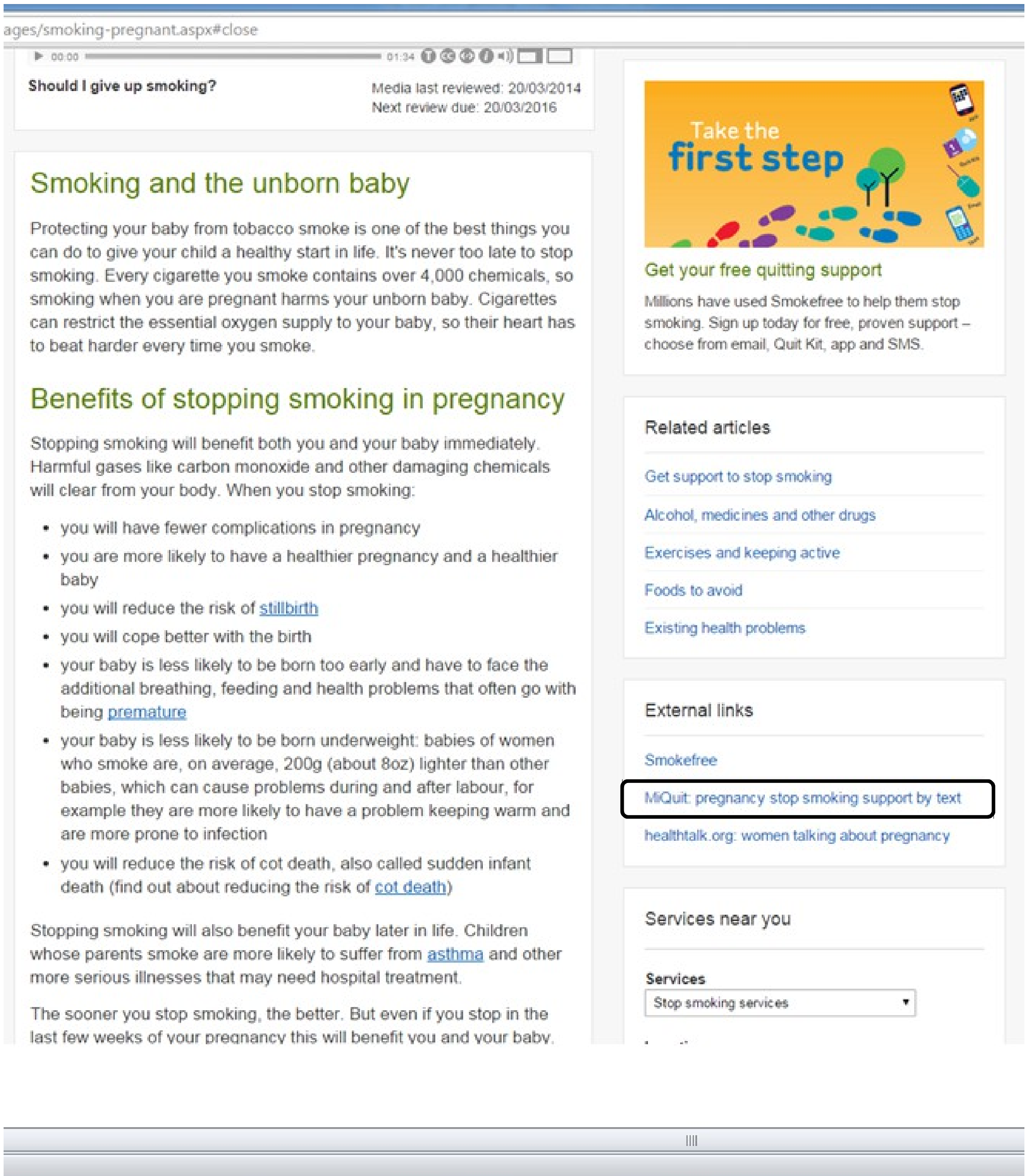

National Childbirth Trust ("Smoking During Pregnancy" Web page)

NCT placed the following text-only link to the MiQuit website under a "Further information" header situated towards the bottom of their "Smoking During Pregnancy" Web page. The link appeared as below (black border added).

- you're more likely to have a healthier pregnancy and a healthier baby and have fewer complications in pregnancy
- you are likely to cope better with the birth
- you have a reduced risk of stillbirth
- your baby may cope better with any birth complication
- your baby is less likely to be born underweight and have problems keeping warm.
- your baby is less likely to be born too early and have the extra breathing, feeding and health problems which often go with prematurity.

The first few days without cigarettes may not be much fun, but the symptoms are a sign your body is starting to recover. You can think about the reasons you stopped, the money you're saving or how much you're helping your baby. Some women find looking at the picture of their baby on the scan or talking to their baby can help when going through tough times and withdrawal symptoms.

Secondhand smoke and pregnancy

If your partner or anyone else in your house smokes, their smoke can affect you and the baby both before and after birth. You are also likely to find it's more difficult to quit. Secondhand smoke can cause low birth weight and cot death. Babies whose parents smoke are more likely to be admitted to hospital with respiratory infections such as bronchitis and pneumonia.

Further information

NCT's helpline offers practical and emotional support in all areas of pregnancy, birth and early parenthood: 0300 330 0700. We also offer antenatal courses which are a great way to find out more about birth, labour and life with a new baby.

Smokefree offers NHS information on smoking in pregnancy including a pregnancy support DVD, cost calculators, 'stressbuster for the mind' and 'stress-buster for the body' MP3 downloads and a Quit app to help with support and encouragement. There is also new NHS-funded support available through MiQuit - text message help with stopping smoking in pregnancy - which provides free and personalised advice, non-judgmental encouragement and information sent direct to your mobile phone.

Smokefree also offers information specifically for fathers.

NHS Pregnancy Smoking helpline is on 0800 123 1044. The helpline is open Mon to Fri 9am to 8pm and Sat and Sun 11am to 5pm. You can also sign up to receive ongoing advice and support at a time that is convenient for you.

QUIT is the UK charity that helps smokers to stop and young people to never start. Information on smoking in pregnancy is available as is information specifically for young smokers.

Quitbecause offers information specifically for young smokers.

In June 2010, NICE published public health guidance on quitting smoking in pregnancy and following childbirth. The guidance How to stop smoking in pregnancy and following childbirth guidance updates recommendations on smoking in NICE's clinical guideline on antenatal care.
